# Gait asymmetry in *glucocerebrosidase* mutation carriers with Parkinson’s disease

**DOI:** 10.1371/journal.pone.0226494

**Published:** 2020-01-24

**Authors:** Anjali Gera, Joan A. O’Keefe, Bichun Ouyang, Yuanqing Liu, Samantha Ruehl, Mark Buder, Jessica Joyce, Nicolette Purcell, Gian Pal

**Affiliations:** 1 Department of Neurological Sciences, Rush University, Chicago, Illinois, United States of America; 2 Cell & Molecular Medicine, Rush University, Chicago, Illinois, United States of America; Emory University, UNITED STATES

## Abstract

**Background:**

*GBA* mutation carriers with PD (PD-*GBA*) are at higher risk of cognitive decline, but there is limited data regarding whether there are differences in gait dysfunction between *GBA* mutation and non-mutation carriers with PD.

**Objectives/Methods:**

The primary aim of this study was to use quantitative inertial sensor-based gait analysis to compare gait asymmetry in 17 PD-*GBA* subjects, 17 non-mutation carriers with PD, and 15 healthy control subjects using parameters that had gait laterality and were markers of bradykinesia, in particular arm swing velocity and arm swing range of motion and stride length.

**Results:**

Arm swing velocity was more symmetric in PD-*GBA* subjects vs. non-mutation carriers in the OFF state (12.5 +/- 8.3 vs. 22.9 +/- 11.8%, respectively, p = 0.018). In the ON-medication state, non-mutation carriers with PD, but not PD-*GBA* subjects, exhibited arm swing velocity (16.8 +/- 8.6 vs. 22.9 +/- 11.8%, p = 0.006) and arm range of motion (26.7 +/- 16.3 vs. 33.4 +/- 18.6%, p = 0.02) that was more asymmetric compared with the OFF-medication state.

**Conclusions:**

In the OFF medication state, arm swing velocity asymmetry may be a useful parameter in helping to distinguish GBA mutation carriers with PD from non-mutation carriers.

## Introduction

Parkinson’s disease (PD) is a neurodegenerative state affecting at least 1 million people in the United States [1]. PD patients differ in their degree of motor disability, rate of progression, response to medication, and level of cognitive impairment. This variation in phenotype is likely due to a combination of specific genetic mutations with superimposed environmental and epistatic contributions [2]. The most common known genetic risk factor for PD is the presence of a mutation(s) in the *glucocerebrosidase* (*GBA*) gene [3].

*Glucocerebrosidase* is a lysosomal enzyme encoded by the beta-glucosylceramidase gene. Approximately 3 to 6% of the general PD population carries a *GBA* mutation, and mutations are more prevalent in those with age of onset of less than 60 years [4]. *GBA* mutation carriers with PD (PD-*GBA*) are at higher risk of cognitive decline [5], but the specific motor phenotype is not well characterized and reports are conflicting. On clinical motor examination, those with PD-*GBA* have a robust Levodopa response [6], are reported to be indistinguishable from non-mutation carriers with PD [7–9]. However, Sidransky et al. [3] reported that PD-*GBA* subjects are more likely to have symmetric motor impairment compared to non-mutation carriers with PD based on Unified Parkinson’s Disease Rating Scale (UPDRS) scores. Davis et al. [10] reported that PD-*GBA* mutation carriers are more likely to have a phenotype that is more consistent with postural instability and gait difficulty (PIGD) [10]. Therefore, our objective was to compare specific a prior defined measures of gait, particularly gait asymmetry, in PD-*GBA*, non-mutation carriers with PD, and healthy controls using quantitative motion assessments. This is of critical importance as large international multi-center clinical trials are ongoing to modify disease progression specifically in PD-*GBA* subjects (NCT02906020). Linking genotype with phenotypic gait markers may allow for detection of changes in motor function with higher sensitivity than traditional clinical rating scales [11], thereby increasingly the likelihood of signal detection in such large clinical trials.

The primary aim of this pilot study was to compare gait asymmetry in the OFF-medication state in PD-*GBA* vs. non-mutation carriers with PD, and healthy controls using quantitative motion analysis. Given previous findings [3], we hypothesized that PD-*GBA* subjects would have more symmetric gait impairment compared with non-mutation carriers with PD in measures of arm swing velocity, arm swing range of motion, and stride length.

These three parameters of gait were chosen because they (1) have laterality and therefore allow us to study symmetry (2) are established markers of bradykinesia [12], and all three parameters are particularly Levodopa responsive in PD [13]. Furthermore, arm swing velocity asymmetry is a parameter that has been well demonstrated in differentiating PD from control subjects using quantitative motion analysis [14]. Arm swing range of motion is the most responsive parameter to Levodopa in the APDM Mobility lab system [13]. Lastly, stride length has been shown to be a strong predictor of changes in the Unified Parkinson’s Disease Rating Scale (UPDRS) Part III [12] and has been shown to have a significant inverse correlation with PD severity [15]. By using this hypothesis-driven approach and choosing only specific measures of gait that were of interest, we were able to examine between group differences with a smaller sample size. The OFF-medication state was chosen to establish the baseline level of bradykinesia and its symmetry without the influence of dopaminergic treatment. We also compared gait asymmetry in the ON-medication state in PD-*GBA* vs. non-mutation carriers with PD to determine whether there are potential differential responses to medication between the two PD groups. To our knowledge, no studies have examined gait asymmetry or other gait parameters in the OFF vs. ON state in PD-*GBA* subjects.

## Methods

Approval for the study was obtained from the Rush University Medical Center Institutional Review Board and all subjects signed written informed consent for study participation. The subjects were recruited based on convenience sampling from the Rush Movement Disorders clinic between July 2016 and March 2019. PD subjects were recruited if they carried a clinical diagnosis of PD by United Kingdom Parkinson Disease Society Brain Bank criteria [16], had age of onset less than 60, were between Hoehn & Yahr stage 1–3 on symptomatic treatment, and were able to ambulate independently without any assistance for 2 minutes in the OFF and ON state. Healthy control (HC) subjects were matched with PD subjects based on age and sex. Subjects requiring assistive walking devices, on treatment with medications that may induce parkinsonism (metoclopramide, typical, or atypical antipsychotic agents), or those with deep brain stimulation were excluded.

### Genetic testing

PD subjects who met inclusion criteria were screened for *GBA* mutation status. Blood samples were sent to the University of Cincinnati Biobank for molecular testing and sequenced for all *GBA* mutations as previously described by the Nichols lab [17,18]. Briefly, the specifics of *GBA* gene sequencing involve using PCR and sequencing primers that were designed as previously described to code exons of the *GBA* gene [17]. The primers were designed to enable preferential amplification of *GBA* over the *GBA* pseudogene also located on chromosome 1. Typically, 60 ng of each genomic DNA is PCR amplified in a 40 ul reaction using conditions empirically determined for each primer pair as previously described [18]. The resulting PCR products for each *GBA* exon are cleaned using 5 ul diluted ExoSAP-IT (Affymetrix, Santa Clara, CA). Fragments are incubated with ExoSAP-IT at 37 degrees for 30 minutes, followed by enzyme inactivation at 80 degrees for 15 minutes. The cleaned PCR products are sequenced on an ABI 3730-XL DNA Analyzer using the BigDye^®^ Terminator v1.1 Cycle Sequencing Kit (Applied Biosystems, Waltham, MA). The resulting DNA sequences are aligned and analyzed using Mutation Surveyor (SoftGenetics, LLC., State College, PA) to identify any potential sequence changes. We report the *GBA* mutations based on severity as variant, mild, and severe mutations. This is important as studies have shown that phenotype, particularly risk for dementia, is strongly modulated by the type of mutation [19].

Non-mutation carriers were matched to *GBA* mutation carriers based on age of onset (+/- 5 years), disease duration (+/- 5 years), and sex. Study staff and subjects were blinded to mutation status at the time of assessment.

### Study design and gait assessment

Demographic data and medical history were collected from all subjects. Subjects with PD provided a list of their parkinsonian medications and current dosing regimen and the MDS-UPDRS part III was completed in the ON-medication state.

At Visit 1 (month 0), all subjects with PD underwent a blood draw for determination of *GBA* mutation status. Visit 2 took place 4 to 6 months later and lasted approximately 90 minutes. PD subjects began their evaluation in the OFF-medication state, with their last dose of dopaminergic therapy taken at least 12 hours prior to testing. They were administered the Instrumented Stand and Walk (I-SAW) protocol utilizing the APDM^™^ Mobility Lab system (APDM Inc) [13]. In this protocol, six inertial sensors were placed on subjects: both wrists, dorsum of both feet, the sternum, and at approximately the fifth lumbar level attached by elastic Velcro straps. The APDM^™^ Mobility Lab software was used to extract all gait and balance parameters. The I-SAW protocol consisted of standing quietly for 30 seconds with hands on hips, followed by an auditory cue to initiate gait, walk seven meters, turn 180 degrees after crossing a marker on the ground, and return to the initial starting point. A template was used to achieve consistent foot placement with 10 cm between the heels and a 30-degree outward rotation of the feet. Three trials of this protocol were completed. Subjects then took their medication and rested for approximately 45 to 60 minutes until they were in their ON medication state. Subjects also verbally verified that they were in their maximal “ON” condition. Three trials of the I-SAW protocol were then repeated in the ON medication condition.

The primary outcome measured was gait asymmetry in PD-*GBA* mutation and non-mutation carriers in the OFF-medication state. Gait asymmetry was measured using three parameters: 1) arm swing velocity (maximum rotational velocity of arm swing; degrees/s), 2) arm range of motion (angular range of arm swing; degrees), and 3) stride length (m). After measuring these parameters, we calculated an asymmetry percentage. For instance, arm swing asymmetry was measured using the formula |(arm swing_left_–arm swing_right_)/(arm swing_left_ + arm swing_right_)| x 100%, as previously described [14] and is reported as a percentage. Using the same formula for arm swing range of motion asymmetry and stride length asymmetry, we reported the respective percentages for these additional measures. A higher percentage value indicates a greater degree of gait asymmetry. Similar analyses were done for subjects in the ON medication state.

To examine the specific gait parameters in more detail, we compared these parameters for the most and least affected sides in the in PD-*GBA*, non-*GBA* subjects (OFF condition), and healthy controls.

### Statistical analysis

Statistical analysis was performed using SAS 9.3. Demographics, clinical variables and gait parameters of PD-*GBA* subjects (reported in percentages), non-mutation carriers, and HCs were compared with an ANOVA test (for normally distributed, continuous variables) followed by post-hoc pairwise comparisons with Bonferroni correction, and Chi-square or Fisher’s exact test (for categorical variables). MDS-UPDRS scores and stride length asymmetry (reported as percentage) were not normally distributed. Therefore, these measures were compared using the Kruskal Wallis test followed by post-hoc pairwise comparisons with Dwass-Steel-Critchlow-Fligner (DSCF) procedure. Paired t-tests were used to compare the three gait parameters in the ON vs. OFF condition within PD-*GBA* subjects and non-mutation carriers with PD. Given the pilot nature of the study and our planned comparisons of only three gait parameters, we did not correct for multiple comparisons [20].

Receiver operating characteristics (ROC) curve analysis was performed to evaluate the discriminatory ability of the gait measures to distinguish PD-*GBA* from non-*GBA* subjects with PD in the OFF state. We analyzed the gait parameters that showed a significant difference between PD-*GBA* and non-*GBA* subjects. Area under the curve (AUC) with 95% confidence interval was computed for relevant measures. Sensitivity and specificity were calculated for the optimal cut-off value using the maximum Youden index.

## Results

### Clinical characteristics

Demographic characteristics of PD-*GBA* subjects, non-mutation carriers with PD, and HCs are shown in Table 1. Age of onset was earlier in PD-*GBA* subjects compared to non-mutation carriers with PD (p = 0.025). In the OFF state, age of onset was not related to the three primary outcome measures based on Pearson correlation analyses (age of onset and arm swing velocity asymmetry: r = 0.13, p = 0.45; age of onset and stride length asymmetry: r = 0.15, p = 0.41; age of onset and arm ROM asymmetry: r = 0.10, p = 0.58). A significant difference was found in MDS-UPDRS Part I scores in non-mutation carriers vs. HCs (p = 0.0003) and PD-*GBA* subjects vs. HCs (p = 0.0001) but were not different between PD-*GBA* vs. non-mutation carriers. Similarly, a significant difference was found in MDS-UPDRS Part II scores in non-mutation and PD-*GBA* subjects vs. HCs (p < 0.0001), but Part II scores were not different between PD-*GBA* subjects vs. non-mutation carriers. (Table 1). Motor scores (MDS-UPDRS Part III) in the ON medication state were significantly worse in both non-mutation carriers and PD-*GBA* subjects vs. HCs, but were not different between PD-*GBA* subjects vs. non-mutation carriers.

**Table 1:** Demographics and baseline clinical data of subjects.

	PD-*GBA* (n = 17)	non-mutation carriers (n = 17)	Healthy controls (n = 15)	p-value
**Age**	57.2 ± 7.4	60.9 ± 5.8	62.7 ± 8.5	0.108
**Age of onset**	46.0 ± 7.8	52.1 ± 7.4	N/A	0.025
**Male (%)**	53	76	47	0.250
**Disease duration**	11.2 ± 6.9	8.8 ± 7.1	N/A	0.326
**H&Y**	2.1 ± 0.5	1.9 ± 0.3	N/A	0.194
**Ashkenazi Jewish, n (%)**	14 (39)	12 (33)	10 (28)	0.434
**Race, n (%)**				
**Caucasian**	15 (88.2)	17 (100)	12 (86)	0.052
**African American**	1 (6)	0	0	
**Other**	0	0	2 (14)	
**Not Provided**	1 (6)	0	0 (n = 14)	
**LEDD**	775.1 ± 422.2	863.6 ± 700.2	N/A	0.659
**MDS-UPDRS Part I**	6.0 ± 4	5.0 ± 4	1.0 ± 1^a^*^b^** (n = 13)	<0.0001
**MDS-UPDRS Part II**	8.0 ± 9	7.0 ± 7	0^a^**^b^** (n = 13)	<0.0001
**MDS-UPDRS Part III**	29.0 ± 21	23.0 ± 14.5	0^a^**^b^** (n = 13)	<0.0001
**MDS-UPDRS Part IV**	0 ± 2	0	0 (n = 13)	0.120

^**a**^: significantly different from *GBA* carriers;

^**b**^: significantly different from non-*GBA* carriers;

*p ≤ 0.0003

**p ≤ 0.0001. *GBA = glucocerebrosidase*

### *GBA* mutation status

**Fig 1:**
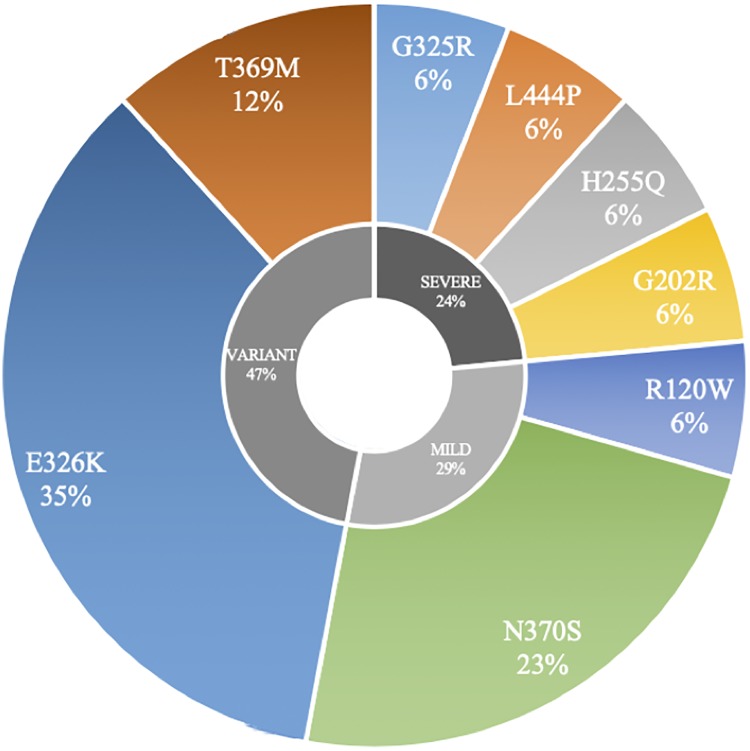
*GBA* mutation type and severity. The distribution of *GBA* mutations classified as risk variant, mild, and severe mutations.

The composition of the three groups and specific *GBA* mutation type and severity are shown in Fig 1. The majority of PD-*GBA* subjects were found to have the E326K mutation (35% of mutation carriers), followed by the N370S mutation (23% of mutation carriers) (Fig 1).

### Motion analysis results

#### Gait asymmetry

Motion analysis results are presented in Table 2 for 17 PD-*GBA* subjects, 17 non-mutation carriers with PD, and 15 HCs. Arm swing velocity was more symmetric in PD-*GBA* subjects vs. non-mutation carriers in the OFF state (12.5 ± 8.3% vs. 22.9 ±11.8%, respectively, p = 0.018), but not in the ON state. Arm range of motion asymmetry and stride length asymmetry were not significantly different between PD-*GBA* subjects vs. non-mutation carriers in the OFF state or ON state.

**Table 2:** Gait asymmetry PD-*GBA* vs. non-*GBA* in the OFF and ON conditions compared with healthy controls.

		OFF medication			ON medication		
	HC	PD-*GBA*	Non-mutation carriers	PD-*GBA* vs. non-mutation carriers, p	PD-*GBA*	Non-mutation carriers	PD-*GBA* vs. non-mutation carriers, p
**Arm swing velocity asymmetry (%)**	12.7 ± 11.3	12.5 ± 8.3	22.9 ± 11.8	0.018	12.2 ± 6.9	16.8 ± 8.6	0.426
**Arm range of motion asymmetry (%)**	14.3 ± 14.4	21.7 ± 15.1	33.3 ± 18.6	0.122	18.9 ± 15.6	26.7 ± 16.4	0.450
**Stride length asymmetry (%)**	2.7 ± 2.5	1.4 ± 1.7	2.6 ± 2.1	>0.05	2.4±1.6	2.7 ± 1.4	>0.05

Gait asymmetry PD-*GBA* vs. non-*GBA* in the OFF and ON conditions compared with healthy controls

GBA = glucocerebrosidase

**Fig 2:**
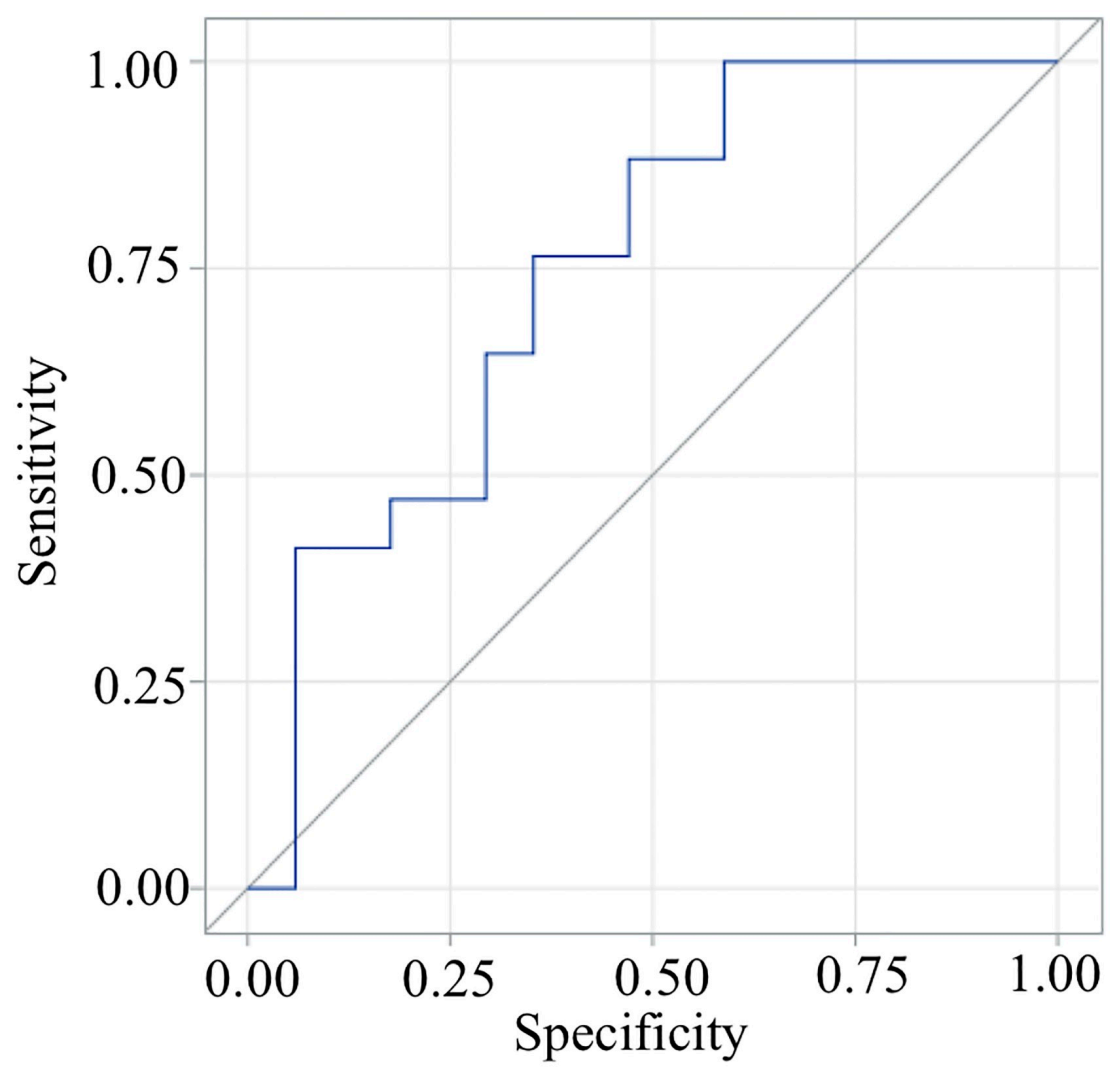
Area under the curve and ROC curve for arm swing velocity asymmetry. Sensitivity, specificity, and receiver operating characteristics (ROC) curve for arm swing velocity asymmetry to distinguish *GBA* from non-*GBA* were completed in the OFF state.

Sensitivity, specificity, and ROC curves for arm swing velocity asymmetry to distinguish PD-*GBA* from non-*GBA* subjects were completed in the OFF state (Fig 2). Area under the curve (AUC) in ROC analysis for arm swing velocity asymmetry in the OFF state was 0.75 (CI: 0.58–0.92). At a cut off value of 17.4% or below, the sensitivity is 0.76 and specificity is 0.65 for arm swing velocity asymmetry.

#### Limb bradykinesia

Arm range of motion for the most affected side was significantly reduced in the non-*GBA* mutation carriers with PD compared to the PD-*GBA* subjects in the OFF state (Table 3). However, arm swing velocity and stride length were not different between the 2 groups on the OFF state.

**Table 3:** Comparison of gait parameters for the most and least affected sides in the in PD-*GBA* and non-*GBA* in the OFF-medication condition and healthy controls.

		PD-*GBA*	Non-mutation carriers	Healthy Controls	p-value
**Arm swing velocity (degrees/s)**	**Most affected**	156.6 ± 62.2	110.9 ± 69.7	168.6 ± 48.4	0.059^+^
	**Least affected**	155 ± 38.8	140.5 ± 38.7		0.299^+^
	**p-value**	0.929	0.148		
**Arm range of motion (degrees)**	**Most affected**	31.4 ± 15.4	15.7 ± 7.1	39.8 ± 15.0	0.001^+^
	**Least affected**	33.1 ± 11.6	29.7 ± 12.8		0.430^+^
	**p-value**	0.725	0.001		
**Stride length (m)**	**Most affected**	1.1 ± 0.1	1.0 ± 0.18	1.2 ± 0.11	0.406^+^
	**Least affected**	1.1 ± 0.11	1.0 ± 0.2		0.060^+^
	**p-value**	0.508	0.630		

Gait parameters and their asymmetry in PD-*GBA* vs. non-*GBA* subjects for most and least affected sides in the OFF state. Mean of both body sides were calculated in controls.

^+^:p-values are comparing *GBA* carriers and non-*GBA* carriers; *GBA = glucocerebrosidase*.

#### Changes in gait symmetry with Levodopa

**Fig 3:**
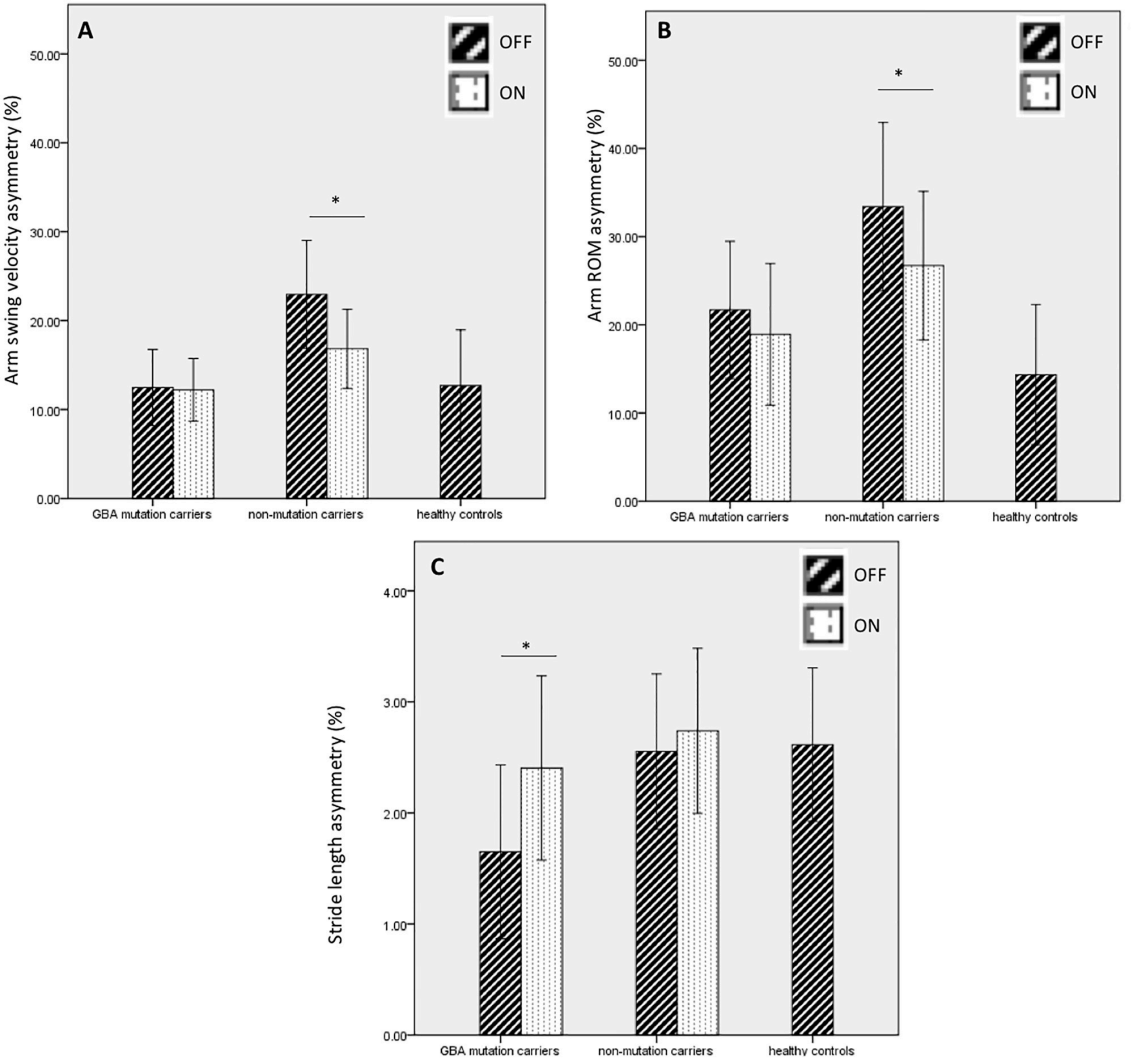
Gait asymmetry comparing PD-*GBA* subjects and non-mutation carriers in the OFF and ON medication state with healthy controls. Arm swing velocity asymmetry (A) arm range of motion asymmetry (B) and stride length asymmetry (C) in the OFF vs. ON states for *GBA* mutation carriers, non-mutation carriers, and healthy control subjects. * p < 0.05.

In PD-*GBA* subjects, stride length was more symmetric in the OFF vs. ON medication state (p = 0.02) (Fig 3). There were no significant differences in arm swing velocity asymmetry or arm range of motion asymmetry in the OFF vs. ON medication state in PD-*GBA* subjects (Fig 3). In non-mutation carriers with PD, arm swing velocity (22.9 ± 11.8 vs. 16.8 ± 8.6, p = 0.006) and arm range of motion (33.3 ± 18.6 vs. 26.7 ± 16.4, p = 0.02) were less symmetric in the OFF vs. ON medication state. There were no significant differences in stride length asymmetry in the OFF vs. ON medication state in this group.

## Discussion

To our knowledge, this is the first pilot study examining the influence of *GBA* mutation status on gait asymmetry using quantitative motion analysis. Our results indicate that PD-*GBA* subjects are more likely to have symmetric arm swing velocity compared with non-mutation carriers with PD in the OFF condition. We did not observe more severe disease in our cohort of PD-*GBA* subjects with regards to the appendicular symptoms we investigated a priori. In fact, arm range of motion on the most affected side was greater in PD-*GBA* subjects compared to non-mutation carriers with PD indicating less severe bradykinesia in the OFF state. This may be due to the fact that nearly half of our PD-*GBA* subjects (47%) had mutations that were classified as risk variants (E326K and T369M). Carriers of these risk variants have a more severe motor and cognitive phenotype than non-mutation carriers, but a *less* severe motor and cognitive phenotype compared with severe mutation carriers (i.e. L444P) [10,19]. Only 24% of our subjects had severe mutations, which may explain why we did not appreciate more severe motor disease in our *GBA* cohort compared with non-mutation carriers. Furthermore, it is possible that appendicular symptoms are less severe in PD-*GBA* carriers and that the more severe phenotype seen in *GBA* mutation carriers reported by previous studies [10] is driven by the presence of more prominent axial symptoms. Future studies should focus on this issue.

In the ON state, we did not find significant differences between PD-*GBA* and non-mutation carriers using either quantitative gait analysis for gait asymmetry in the 3 parameters investigated or in MDS-UPDRS motor scores. This is consistent with other studies that have reported no clear motor difference between *GBA* and non-*GBA* subjects in the ON-medication state [21]. Our study suggests that subjects should be examined in the OFF-medication state to potentially observe a difference in phenotype which may be particularly useful for clinical trials focused on disease modification in PD-*GBA* subjects [22]. Furthermore, our study demonstrates that specific aspects of gait are differentially affected by Levodopa based on *GBA* mutation status. For instance, in non-mutation carriers with PD, but not PD-*GBA* subjects, arm swing velocity asymmetry and arm range of motion asymmetry improved to become comparable to that seen in healthy control subjects.

The mechanism underlying a more symmetric motor phenotype in *GBA* mutation vs. non-*GBA* mutation carriers is unknown. Studies using animal models and in vitro pluripotent stem cell technology modeling PD disease have demonstrated increased and more widespread alpha-synuclein distribution and aggregation in the setting of loss of function in the *glucocerebrosidase* enzyme [22–25]. Low *glucocerebrosidase* enzyme activity can lead to buildup of glucosylceramide in lysosomes which in turn accelerates the formation of alpha-synuclein aggregation [25, 26]. Therefore, it is possible that low *glucocerebrosidase* activity leads to the more widespread and diffuse distribution of alpha-synuclein, resulting in a more symmetric motor phenotype. However, imaging studies have shown that *GBA* mutation carriers display a greater striatal asymmetry index than those with sporadic PD and PD subjects with alpha-synuclein, PINK1, and Parkin mutations [27]. We posit that striatal asymmetry may not be the sole factor responsible for the motor phenotype of *GBA* mutation carriers. For example, PD-*GBA* carriers have been shown to have lower fluorodeoxyglucose uptake in bilateral parieto-occipital association areas resulting in visuospatial dysfunction [28]. The combination of striatal dysfunction and bilateral visuospatial impairment may be responsible for the more symmetric phenotype seen in our study. In addition, it is possible that other locomotor networks such as those in the pedunculopontine nucleus are differentially affected in *GBA* vs. non-*GBA* mutation carriers. Further work is needed to investigate these hypotheses.

Our findings indicate that *GBA* mutation carriers are likely to have more symmetric arm swing velocity compared to non-mutation carriers. Arm swing velocity asymmetry may be useful in combination with other described features of *GBA* mutation carriers such as earlier age of onset and more severe olfactory dysfunction, rapid eye movement sleep behavior disorder, and visuospatial dysfunction [29] to contribute to the targeted screening and identification of *GBA* mutation carriers in both research and clinical practice. Indeed, as clinical trials are ongoing for *GBA* mutation carriers with PD [30, 31], genotype-phenotype correlations may be useful in identifying individuals at high risk of carrying mutations in the *GBA* gene in order for these clinical trials to be cost-effective and successful, as well an in selecting the most appropriate outcome measures.

The strengths of our study include being the first study that examines the influence of *GBA* mutation status on gait asymmetry using quantitative motion analysis and this was performed in both the OFF and ON medication condition. Furthermore, we used the APDM Mobility Lab motion analysis system, which provides portable, validated, objective, and reliable measures of gait that are sensitive to PD [32]. Also, our study is hypothesis driven and only the parameters relevant to our hypothesis were chosen. However, our study has several limitations. While our study assessed MDS-UPDRS scores in the ON state, these were not completed in the OFF state in all qualifying PD subjects as we wanted to limit the testing burden. Therefore, we are unable to determine whether we would have found differences between PD-*GBA* subjects vs. non-mutation carriers in the OFF state using the MDS-UPDRS. Furthermore, given our small sample size of GBA mutation carriers with severe mutations, we were unable to draw any conclusions regarding the influence of mutation severity on gait asymmetry. Future studies with a larger sample size would allow for comparison of asymmetry parameters based on *GBA* mutation severity. However, these studies will likely need to multi-center collaborations with standardized procedures given the burden of testing associated with quantitative motion analysis and large number of subjects that need to be screened and matched to perform such analyses.

Finally, differences in balance, bradykinesia (examining the most affected side), and the influence of cognition on gait and balance comparing PD-*GBA* subjects to non-mutation carriers with PD remain to be examined in future studies.

## Supporting information

S1 File Raw data for subjects OFF medication. This is the raw gait data for all subjects OFF medication.
(SAV)

S2 File Raw data for subjects ON medication. This is the raw gait data for all subjects ON medication.
(SAV)
